# Prevention of non-response to cardiac resynchronization therapy: points to remember

**DOI:** 10.1007/s10741-019-09834-w

**Published:** 2019-07-27

**Authors:** Huolan Zhu, Tong Zou, You Zhong, Chenguang Yang, Yirong Ren, Fang Wang

**Affiliations:** 1 Department of Cardiology, Beijing Hospital, National Center of Gerontology, Dahua Road No.1, Beijing, 100730 People’s Republic of China; 2 Peking Union Medical College, Chinese Academy of Medical Science, Beijing, People’s Republic of China

**Keywords:** Cardiac synchronization, Non-response, Patient selection, Electrode implantation, Program control optimization

## Abstract

Cardiac resynchronization therapy (CRT) is an important and effective therapy for end-stage heart failure. Non-response to CRT is one of the main obstacles to its application in clinical practice. There is no uniform consensus or definition of CRT “response.” Clinical symptoms, ventricular remodeling indices, and cardiovascular events have been reported to be associated with non-responders. To prevent non-response to CRT, three aspects should be thoroughly considered: preoperative patient selection, electrode implantation, and postoperative management. Preoperative selection of appropriate patients for CRT treatment is an important step in preventing non-response. Currently, the CRT inclusion criteria are mainly based on the morphology of QRS waves in deciding ventricular dyssynchrony. Echocardiography and cardiac magnetic resonance are being explored to predict nonresponse to CRT. The location of left ventricular electrode implantation is a current hot spot of research; it is important to identify the location of the latest exciting ventricular segment and avoid scars. Cardiac magnetic resonance and ultrasonic spot tracking are being progressively developed in this field. Some new techniques such as His Bundle pacing, endocardial electrodes, and novel sensors are also being investigated. Postoperative management of patients is another essential step towards preventing non-response; it mainly focuses on the treatment of the disease itself and CRT program control optimization. CRT treatment is just one part of the overall treatment of heart failure, and multidisciplinary efforts are needed to improve the overall outcome.

## Introduction

Cardiac resynchronization therapy (CRT) is an important non-pharmacological therapy in end-stage heart failure. It is effective in improving the symptoms, reversing ventricular remodeling, and reducing mortality. However, it is not yet ideal for use because of two main reasons: high cost and approximately 30% non-response rate. Even though many patients meet the inclusion criteria for CRT implantation, not all of them develop a response. Non-response is due to multiple perioperative factors and to overcome them, combined efforts of multiple disciplines are required. Prevention of the incidence of nonresponse is a pivotal step in improving the overall efficacy of CRT treatment [1, 2].

## The definition of nonresponse

Although CRT has been developed for over 20 years, there are no clear and uniform definitions or consensus defining response and non-response in CRT. In current clinical practice or trials, the response can be determined from three aspects: clinical event criteria, evaluation criteria of myocardial remodeling, and evaluation criteria for the composite endpoint [1]. The clinical event criteria includes indicators related to cardiac function such as New York Heart Association (NYHA) classification, cardiopulmonary exercise results, 6-min walking test results, and the quality of life score. Additionally, it includes criteria related to event occurrence such as all-cause mortality and unplanned hospitalization rate [2]. As quantifiable parameters, myocardial remodeling criteria are generally the indices used in large clinical trials. Echocardiography is an important method to assess myocardial remodeling, which is mainly reflected in the volume of the left ventricle. In most clinical trials, non-response to CRT is identified when left ventricular end-systolic volume index decreases [3]. The composite endpoint is also commonly used to evaluate CRT response in clinical trials; multiple landmark clinical trials, such as BLOCKHF and IN-TIME, have demonstrated a complex endpoint for heart failure [3, 4].

Currently, the homogeneity of the results of clinical trials is poor, which has, in turn, seriously impaired further developments in this field. It has been pointed out that of 26 articles with the most cited CRT responses, a total of 17 criteria were used to predict CRT response, and approximately 75% of these criteria were weakly consistent with only % being strongly consistent [5]. The non-response rates were the lowest when the results of the 6-min walk test or quality of life scores were used, and the highest when hard outcomes such as hospitalization and mortality were used [6].

## Preparation for implantation — patient selection

Selection of the right patients for CRT is critical for good prognoses. Before CRT implantation, the patients with the following conditions should be excluded: untreated myocardial ischemia, valvular heart disease, mechanical obstruction, and untreated arrhythmia–induced cardiomyopathy. Electrocardiogram (ECG) is the most important investigation in the selection of patients in order to identify those with ventricular dyssynchrony. The latest European guideline identify complete left bundle branch block (LBBB) with QRS duration ≥ 150 ms as class IA evidence, while QRS duration between 130 and 150 ms is identified as class IB evidence. Patients with QRS duration < 130 ms should not be treated with CRT. Patients with non-left bundle branch block belong to the class II evidence group [7]. In patients with non-left bundle branch conduction block, left ventricular dyssynchrony is often not significant. After the implantation of CRT, the rate of nonresponse is higher in these patients than those with left bundle branch block, with worsening of the clinical condition in some patients [8]. Despite current concerns about the effects of CRT implantation in patients with non-left bundle branch block, recent studies have indicated that the QRS duration in patients with right bundle branch block is significantly shorter and left ventricular function can be improved by permanent His bundle pacing, which may be a promising option for cardiac resynchronization in this subgroup of patients with reduced left ventricular ejection fraction [9]. Better outcomes are usually observed in patients with left bundle branch block with the following features that often suggest left intraventricular conduction delay: lead V1 with QS or rS wave; lead V6 with R wave; leads I, aVL, V5, and V6 with notches on the R wave [10, 11]. The latest studies have reported a strong positive correlation between QRS area and CRT response and that this correlation is as valuable as the other current ECG criteria. QRS area may be predictive of CRT response in patients with non-wide left bundle branch block [12]. In addition to ECG, magnetic resonance and other tests were used in recent studies to determine left ventricular dyssynchrony. A new study indicates that assessment of mechanical dyssynchrony using apical rocking or septal flash technique can improve the prognostic value of guideline-based patient selection for cardiac resynchronization therapy [13]. However, the results from echocardiogram and magnet resonance were inconsistent and not unanimously recognized. Therefore, such methods should not be currently used as standard practices in selecting patients. However, such methods can be evaluated as references for predictions. Newer technologies such as three-dimensional spot tracking technology and stress ultrasound can be used to screen for some characteristics that are related to non-response.

Patients with different clinical features have different outcomes. A previous study reported that ischemic heart disease, male sex, NYHA class IV, severe mitral regurgitation (MR) and left atrial expansion, and shorter ventricular mechanical delay were associated positively with non-response in CRT. However, these features are limited to the process of patient selection and cannot be used as independent predictors of nonresponse [14].

In patients with atrial fibrillation, the efficacy of CRT implantation remains controversial [15]. For heart failure patients with atrial fibrillation, the current recommendation is that radiofrequency catheter ablation plus CRT is more effective than pharmacological therapy [16].

## Operation procedure

### Implantation of right ventricular electrode

Generally, the right ventricular electrode is implanted at the apex. Some researchers have argued that non-apical pacing is more of a physiological mimic than apical pacing. A meta-analysis that evaluated implantation of right ventricular electrodes into the apical and non-apical segments concluded that implantation of electrodes at the interventricular septum and right ventricular outflow tract was more effective in preserving left ventricular function, especially in patients with impaired ejection fraction [17]. However, post hoc analysis in large studies demonstrated no differences in cardiac remodeling and clinical endpoints in patients who underwent CRT irrespective of placement of the right ventricular electrode at the apex or septum [18]. A computer simulation of Bi-ventricular pacing found that the right ventricular upper septum near the outflow tract was an alternative location for the right ventricular apex leads [19]. More clinical trials are needed to accumulate strong evidence regarding the positioning of the right ventricular electrode.

### Left ventricular electrode implantation

The key point for the implantation of left ventricular electrodes is the identification of the latest exciting spot. In most patients with heart failure, the latest exciting segment in the left ventricle is located in the lateral wall or posterior wall; therefore, it is recommended that the left ventricular electrode should be preferentially implanted at either of these locations. However, while the optimal implantation site varies because of the heterogeneity of coronary veins, apical implantation should be avoided [20].

A previous study discovered that ejection fraction (EF), end systolic volume (ESV), end-diastolic volume (EDV), and quality of life (QOL) measurements were most improved at 6 months of follow-up in patients who underwent CRT if the activation delay from the beginning of QRS to the electrode position was more than 95 ms [21]. In recent years, many studies have attempted to identify the latest location of left ventricular activation. Echocardiography—as a non-invasive, easy, and repeatable method—has shown great promise. Another study that used spot tracking technique reported that the nonresponse to CRT could be reduced if the electrodes were placed near the site of the latest activation while avoiding the scar site [22]. However, spot tracking technique is time-consuming and labor-intensive, and the results may vary between operators; therefore, it is currently difficult to implement it in clinical practice. Stress echocardiogram has also been used to determine the myocardial scar; yet, there is no evidence for its effectiveness and safety in large trials [23].

It is crucial to avoid scars while implanting electrodes. Cardiac magnetic resonance (CMR) is the gold standard for evaluating myocardial structure and function because of its unique imaging ability for cardiac scars. Pre-operative magnetic resonance imaging is important for identifying the cause of heart failure and guiding left ventricular electrode implantation away from the myocardial scar. Kenneth et al. and other researchers reported that determination of the scar position using magnetic resonance examination and implantation of the left ventricular electrode while avoiding the scar could significantly reduce the incidence of nonresponse [24, 25]. Bisson suggested that the extension of QRS width was related to the location of the scar, which can be judged with a combination of CMR, computed tomography, and ECG examinations. While this is a simple and effective method to estimate the position of the left ventricular electrode, more data are needed to confirm the same [26].

Because of various reasons, some patients with conventional ventricular pacing do not respond to CRT. Studies on left ventricular double electrode pacing have discovered that myocardial remodeling can be improved. In patients who are non-responsive to biventricular pacing, left ventricular pacing alone may be considered [6]. Additionally, more pacing configurations such as quadrupole electrodes have been used in clinical practice. By using three-dimensional speckle tracking technique, researchers have found that multipole pacing (MPP) can reduce ventricular dyssynchrony index and increase ejection fraction [27]. A randomized controlled clinical trial has demonstrated that quadrupole electrodes can significantly reduce the perioperative complications and improve clinical outcomes [28]. However, the latest study reveals that optimization of the pacing site of a quadripolar LV lead is more important than to program MPP [29].

Electrode implantation with avoidance of scars and choosing the latest exciting site—irrespective of mechanical contraction and electrical activity—is the best approach to ventricular synchronization and can improve the response rate. Some common postoperative reasons for nonresponses include failure in identifying the optimal location, insufficient ventricular resynchronization, and loss of ventricular capture. There are significantly more complications during secondary implantation than there are during the primary implantation; therefore, optimization should be performed immediately after CRT implantation. If synchronization after the implantation surgery is not satisfactory, re-implantation can be considered [1]. Regardless of the place where the left ventricular electrodes are implanted, the ultimate goal is to maximize left ventricular output volume. A new study shows computer simulations capture LV and RV behavior during pacing delay variation and may be used in the design of new CRT optimization studies [30].

### Other new techniques about electrode implantation

Recently, new techniques have focused on recording the myocardial systolic activity and changes in blood flow in the chambers, in order to automatically adjust the atrioventricular (AV) and interventricular (VV) delay accurately. These techniques are tailored to the individual characteristics of patients. Brugada’s study suggested that the sensor SonR embedded in the right atrial electrode could record the blood flow signals and that these signals are related to maximal rate of rise of left ventricular pressure (dP/dtmax). According to the relevant data, the sensor can automatically adjust the AV/VV delay and reduce the incidence of nonresponse [31].

Another current focus is left ventricular endocardial pacing [32]. Theoretically, electrical signals are better transmitted from endocardium to epicardium, than from epicardium to endocardium. Derval et al. found that left ventricular endocardial pacing can result in better hemodynamics [33]. The feasibility and safety of this procedure were investigated in multiple centers previously, with a reported success rate of 90%; however, in the study, 14 transient ischemic attacks (9 patients, 6.8%), 5 non-disabling strokes (5 patients, 3.8%), and 23 deaths (17.4%) were observed [34]. Due to fatal risks such as infective endocarditis, more clinical trials are needed to confirm its safety.

In a head-to-head study, Ahran found that patients’ hemodynamic improved better in His bundle pacing group than the traditional biventricular pacing group [35]. His bundle pacing is an especially promising alternative for patients who were non-responsive to biventricular pacing. In Sharma et al.’s study including 106 patients, they found that His bundle pacing can significantly improve patients’ heart function and ejection fraction [36]. Besides His bundle pacing, another newpacing mode–left bundle branch pacing, has been applied in clinical practice. Results have indicated that the parameters of pacing threshold, sensitivity, and impedance were comparable to those of the traditional biventricular pacing during the 3-month follow-up. Left bundle branch pacing can make QRS width narrower and more closely mimic the human physiology; it is a new and promising method to improve ventricular dyssynchrony [37].

## Post-operation management

Follow-up is vital, including program optimization, treatment of heart failure, and daily rehabilitation. A study showed that 73% of postoperative patients were not managed appropriately, which contributed a lot to the non-response rate [38].

### Program optimization

Electrical synchronization of CRT is mainly determined by the morphology and width of the QRS wave. Several studies have reported that the narrower the width of QRS, the higher is the response rate of CRT [39]. Other studies have reported that V1 with tall R waves often implies better outcomes as well [40]. The most important settings for optimization include the pacing mode, upper and lower rates, capture output, stimulus energy configuration, and AV-VV delay. The upper limit tracking rate must be high enough to allow simultaneous pacing of both chambers when the patient is in motion [41]. Mullens found that inappropriate settings of AV-VV delay can seriously affect the left ventricular flow output, which was one of the important reasons for nonresponse [38]. Doppler ultrasound has become a common method to optimize the AV-VV delay. Although ultrasound optimization of CRT was thought to be ideal, recent large multi-center clinical studies have shown that the results are not promising or even as effective as a clinician’s empirical adjustment of the AV-VV delay. These clinical trials also found that CRT automatic optimization was not superior to empirical manual optimization [42, 43]. It has been reported that in 50% of patients, a part of the measured biventricular pacing during programmed pacing is actually a fusion wave; this phenomenon is more obvious in patients with atrial fibrillation [44]. To solve this problem, a new algorithm has been developed; when the total capacity of fusion pacing exceeds a certain threshold, the pacemaker will automatically increase the pacing frequency appropriately [45]. Such a new algorithm for left ventricular fusion wave pacing may be useful in non-responders. Currently, other optimization algorithms and new methods such as sub-endocardial hemodynamic sensors are being investigated in clinical trials, and the results are awaited for further confirmation [46, 47]. In short, optimization of CRT is still inconclusive. Therefore, clinicians should develop individualized program control optimization strategies for patients who do not respond.

Remote monitoring is a highly recommended technology today. It can help assess the proportions of biventricular pacing, paroxysmal atrial fibrillation, frequent ventricular tachycardia, and other such events that might result in nonresponse, and the treatment can be adjusted any time [48]. Multi-center clinical studies in many countries have found that remote monitoring can reduce the number of visits to the hospital compared with routine follow-up in the consulting room, and it can also help check-in at the hospital on time when an event occurs. IN-TIME study manifested that in 1 year of follow-up, the composite score of clinical deterioration and all-cause mortality reduced significantly with the use of remote monitoring [4]. However, other studies have found no significant reduction in the hospitalization rate or death from cardiovascular events in patients under remote monitoring [49].

### Treatment of heart failure and concomitant diseases

Besides optimization of the parameters, postoperative treatment of the disease itself should also be paid attention to, especially in patients who are non-responsive to CRT. A previous study reported that atrial arrhythmias lasting for more than 10 min were positively associated with nonresponse and mortality [50]. A study in 1838 patients found that sinus rhythm was maintained after radio frequency (RF) ablation for atrial fibrillation in 60% of patients at 23 months of follow-up. Surgical complications were observed in 4.2% patients; LVEF increased by an average of 13% [51]. However, some researchers believe that patients with atrial fibrillation who undergo CRT often have significant EF decline and are therefore unlikely to benefit from RF ablation. There is insufficient data on RF ablation for atrial fibrillation in patients with CRT [1]. A study reported a long-term efficacy rate of 88% in 65 patients with premature ventricular beats greater than 10,000 in 24 h who were non-responsive and underwent RF ablation. At 1-year follow up, LVEF had increased by a mean value of 7% and the left ventricular volume had decreased by a mean value of 18%. Therefore, RF ablation should be considered for non-responders with frequent premature ventricular contractions when medical interventions are invalid [52].

Mitral valve insufficiency is usually alleviated in a large proportion of patients after CRT. If moderate-to-severe mitral valve insufficiency does not change from the baseline value at 6 months after the surgery, it indicates an increased rate of non-response and mortality [53] re-vascularization is recommended for patients with coronary heart disease (CHD) who undergo CRT, if CHD contributes to the heart function. A study found that after re-vascularization, the re-hospitalization, cardiovascular mortality, and all-cause mortality rates had a declining trend. In such patients, more data are needed to confirm whether interventions for CHD have a significant effect on CRT response [48].

In patients who undergo CRT, heart function improves after the implantation. Drugs such as angiotensinogen-converting enzyme inhibitors (ACEI) and beta blockers should be adjusted as required. Appropriate control of chronic heart failure is a complicated and tough task; it needs to be carried out comprehensively, including heart failure education, rehabilitation, and close monitoring.

## Conclusion

Cardiac resynchronization therapy is a vital treatment for end-stage heart failure. It is critical to prevent the incidence of non-response, which requires multidisciplinary and multifaceted efforts. Preoperative patient selection, electrode implantation, and postoperative optimization should be emphasized. Simultaneously, equal attention should be paid to the follow-up, management, and pharmacological treatment of heart failure and its complications in order to provide the ultimate benefit for the patients.
